# Golexanolone Attenuates Neuroinflammation, Fatigue, and Cognitive and Motor Impairment in Diverse Neuroinflammatory Disorders

**DOI:** 10.3390/ph18111757

**Published:** 2025-11-18

**Authors:** Marta Llansola, Gergana Mincheva, Yaiza M. Arenas, Paula Izquierdo-Altarejos, Maria A. Pedrosa, Thomas P. Blackburn, Torbjörn Bäckström, Bruce F. Scharschmidt, Magnus Doverskog, Vicente Felipo

**Affiliations:** 1 Laboratory of Neurobiology, Centro de Investigación Príncipe Felipe, 46012 Valencia, Spain; mllansola@cipf.es (M.L.); givaylova@cipf.es (G.M.); ymarenas@cipf.es (Y.M.A.); pizquierdo@cipf.es (P.I.-A.); mapedrosa@cipf.es (M.A.P.); 2 INCLIVA Instituto de Investigación Sanitaria, 46010 Valencia, Spain; 3 Umecrine Cognition, SE-171 65 Solna, Sweden; thomas.blackburn@umecrine.se (T.P.B.); torbjorn.backstrom@umecrine.se (T.B.); bruce.scharschmidt@umecrine.se (B.F.S.); magnus.doverskog@umecrine.se (M.D.); 4 Department of Clinical Sciences, Obstetrics and Gynecology, Umeå University, SE-901 87 Umea, Sweden

**Keywords:** golexanolone, GABA neurotransmission, microglia activation, neuroinflammation, fatigue, cognitive impairment, motor impairment, hepatic encephalopathy, primary biliary cholestasis, Parkinson’s disease

## Abstract

**Background and Objectives**: Neuroinflammation plays a significant role in liver and neurological disorders via its disruption of neurotransmission, which alters cerebral function, resulting in cognitive and motor impairment, fatigue, anxiety, and depression. A key interaction exists between GABAergic neurotransmission and neuroinflammation, whereby excessive GABA_A_ receptor activation exacerbates cognitive and behavioural impairment. Golexanolone, a novel GABA_A_-receptor-modulating steroid antagonist (GAMSA), primarily attenuates GABAergic potentiation via GABA_A_-positive steroid allosteric receptor modulators such as allopregnanolone. This review aims to summarize new evidence showing that golexanolone improves peripheral inflammation, neuroinflammation, and neurological alterations in animal models of different neurological pathologies. We provide an overview of the first clinical trial using this novel compound. **Results**: In rat models of hyperammonemia and minimal hepatic encephalopathy (MHE), peripheral inflammation induces microglia and astrocyte activation and neuroinflammation, altering GABAergic neurotransmission and resulting in cognitive and motor impairment. Golexanolone’s unique dual action reduces peripheral inflammation and glial activation, thus normalizing neurotransmission and cognitive and motor function. Furthermore, a phase II study in cirrhotic patients with MHE shows that golexanolone is well tolerated and improves cognition. Similarly, in a model of primary biliary cholangitis (PBC) involving bile-duct ligation, peripheral inflammation, neuroinflammation, and altered neurotransmission—associated with fatigue, impaired memory, and locomotor gait and motor incoordination—were reversed by the dual action of golexanolone. In the Parkinson’s disease (PD) rat model induced by neurotoxin 6-OHDA, rats exhibited fatigue, anhedonia, impaired memory, and locomotor gait and motor incoordination, which were associated with microglia and astrocyte activation in the substantia nigra and striatum, in addition to tyrosine hydroxylase (TH) loss. Golexanolone reduces microglia and astrocyte activation, partially reduces TH loss, and improves fatigue, anhedonia, memory, locomotor gait, and motor incoordination. Golexanolone also normalizes elevated levels of α-synuclein. **Conclusions**: These findings suggest that golexanolone has beneficial therapeutic effects for treating fatigue, depression, motor, and cognitive impairment across diverse neuroinflammatory conditions, including synucleinopathies.

## 1. Introduction

### Golexanolone Selectively Reduces GABAergic Neurotransmission

**Figure 1:**
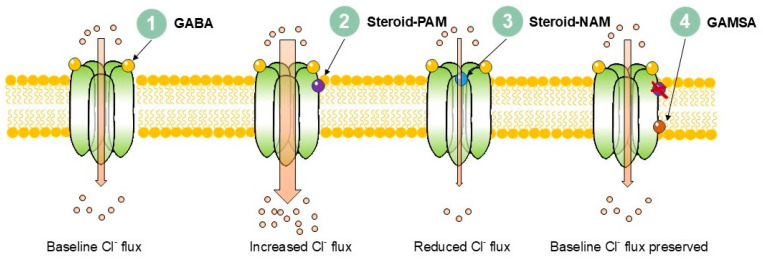
Mechanisms of neurosteroid modulation of GABA_A_ receptor signalling.

Neurosteroids exhibit pleiotropic activities in the central nervous system (CNS) and are important endogenous and synthetic modulators of GABA_A_ receptors, the main inhibitory neurotransmitter system in the brain. Their actions as modulators of GABA_A_ receptors are generally divided into three groups: positive allosteric modulators (steroid-PAMs), negative allosteric modulators (steroid-NAMs), and GABA_A_-receptor-modulating steroid antagonists (GAMSAs). Each group interacts with distinct binding sites on the GABA_A_ receptors and produces characteristic effects [1,2,3] (Figure 1).

1. Baseline: GABA binds to the extracellular domain of the receptor, producing chloride influx. 2. Steroid-PAM: Positive allosteric modulators (e.g., allopregnanolone, THDOC) bind to transmembrane domain pockets and increase chloride influx. 3. Steroid-NAM: Negative allosteric modulators (e.g., pregnenolone sulphate, DHEAS) bind extracellularly close to the chloride conduction pathway and reduce channel activity, decreasing chloride influx. 4. GAMSA: 3β-Hydroxy GABA_A_-receptor-modulating steroid antagonists (e.g., golexanolone) bind at or near canonical neurosteroid PAM sites but with a reversed hydroxyl group orientation and block steroid modulation while preserving normal GABA signalling.

These groups follow clear chemical rules, mainly depending on the orientation of the hydroxyl group at carbon-3 and the presence of sulphate substitutions [4].

The classical steroid-PAMs are 3α-hydroxylated pregnane steroids, such as allopregnanolone and tetrahydrodeoxikortikosteron (THDOC). They work at very low concentrations (nanomolar) to increase GABA-evoked chloride currents by stabilizing the open state of the channel, increasing inhibition. This potentiation affects both phasic and tonic inhibition—depending on the receptor subtype and intra-synaptic or extra-synaptic localization—and underlies sedative, anticonvulsant, anxiolytic, and anesthetic properties [2,5,6]. At higher concentrations, steroid-PAMs can open the channel in the absence of GABA [7]. Molecular studies show that at least two main binding sites in the transmembrane domain can be reached through the lipid bilayer [8,9,10]. Cryo-EM now provides structural evidence for the binding of allopregnanolone, and it is crucial to note that the steroid action comprises a hydrogen bond donor at the C3 position and a hydrogen bond acceptor at the C20 position [11,12,13,14]. The 3α-hydroxyl group is essential for the steroid-PAMs’ potentiation effect, as the 3β-epimer exhibits no steroid-PAM activity [15,16,17,18]. δ-containing extrasynaptic GABA_A_ receptors are especially sensitive, explaining the role for neurosteroids in tonic inhibition [19]. There exist several naturally circulating steroid-PAMs, but the most potent are allopregnanolone, tetrahydrodeoxycorticosterone (THDOC), pregnanolone, and androstanediol [20].

Sulphated steroids, such as pregnenolone sulphate (PS) and dehydroepiandrosterone sulphate (DHEAS), act as steroid-NAMs, while 3β-hydroxy-steroid compounds generally do not. Both, however, reduce GABA currents, increase desensitization by promoting receptor desensitization, and stabilize the closed or desensitized state of the GABA_A_ receptor [18,21,22]. The main difference between steroid-NAM and 3β-hydroxy-steroid compounds is their ability to close and maintain closure of the ion channel in the GABA_A_ receptor; moreover, 3β-hydroxy-steroid compounds are particularly effective when the receptor is already active [21]. Steroid-NAMs’ binding determinants differ from those of steroid-PAMs, and they are located near the extracellular transmembrane portals of the receptor [22,23]. This results in decreased inhibition (disinhibition [24]) and, at the network level, increased excitability. Functionally, some steroid-NAMs have been associated with pro-cognitive, anxiogenic, and occasionally proconvulsant effects [2,21].

The GAMSAs are a unique pharmacological class of compounds that primarily block the effect of steroid-PAMs without disturbing normal GABA function [3,25,26]. Iso-allopregnanolone (3β-epimer of allopregnanolone) was first described as an endogenous GAMSA [3,16,27]. Later, synthetic steroids such as 17-PA were developed, exhibiting the selective reversal of neurosteroid-induced sedation [25,28]. Most recently, golexanolone (GR3027) has entered clinical development, showing promising results in hepatic encephalopathy (HE) and potential applications in Parkinson’s disease (PD) and primary biliary cholangitis (PBC) [29,30,31]. This selectivity renders GAMSAs, such as golexanolone, important tools for both research and therapeutic use in abnormal neurosteroid tone and GABAergic neurotransmission conditions [3,32], including aberrant extrasynaptic tonic inhibition [33], as they can separate steroid modulation from basic GABAergic transmission.

Multiple disease states show a shift in endogenous neurosteroid tone at GABA_A_ receptors: 3α-steroid PAMs such as allopregnanolone and THDOC are increased or overactive, whereas sulphated and 3β-hydroxy steroid-NAMs such as pregnenolone sulphate and DHEAS are decreased. This imbalance biases neural networks toward excess GABAergic inhibition, impairing several behavioural symptoms, such as wakefulness, attention, and cognition. HE in patients with cirrhosis and PBC (autoimmune cholestasis) are two liver-related inflammatory diseases that show elevated brain allopregnanolone and related steroid-PAMs, correlating with impaired consciousness and cognition [34,35]. Luchetti and co-workers [36] reported that the levels of allopregnanolone were disease-stage dependent in patients with PD and were increased during the earlier PD-Braak stages 1-4 (PD4), while they were diminished in PD-Braak stage 6 (PD6) compared to PD4. In PD patients with heterozygous glucocerebrosidase (GBA) mutations (GBA-PD), Cavallieri and co-workers [37] found that allopregnanolone levels correlated with a more severe motor impairment, and they were negatively correlated with cognitive dysfunction. Several disorders also display reduced levels of sulphated and 3β-hydroxy neurosteroids, such as in Alzheimer’s disease [38], Schizophrenia [39], PD [40], major depressive disorder, MDD [41], PBC [35], and HE [42]. In PBC, reduced DHEAS correlated with fatigue severity. Interestingly, exogenous DHEAS was shown to improve cholestasis-associated fatigue in bile-duct-ligated rats, as a model of cholestasis in PBC [43], and pregenolone sulphate exerts antidepressant-like effects [44].

Neurosteroids are pleiotropic molecules acting on several molecular targets [45] and are involved in various neurodegenerative diseases associated with neuroinflammation [46]. GAMSAs such as golexanolone primarily antagonize steroid-PAM’s enhancement of GABA_A_ currents [47] without blocking GABA itself. In diseases with increased steroid-PAMs and decreased steroid-NAMs, GAMSA therapy can thus be viewed as a physiological replacement of the lost counter-modulatory tone, normalizing neurosteroid-enhanced inhibition and improving arousal, cognition, and wakefulness without blocking baseline GABA signalling.

Recent studies have highlighted a remarkable interplay between neuroinflammation and the enhancement of GABAergic neurotransmission in different pathological situations. Here, we summarize the evidence and mechanisms involved in the effects of golexanolone on inflammation and neuroinflammation and neurological impairment in three neurological pathologies in which neuroinflammation is a main pathological hallmark: minimal HE (MHE), PBC, and PD.

## 2. Golexanolone Reduces Peripheral Inflammation, Neuroinflammation, and Cognitive and Motor Impairment in Hyperammonemia and Minimal Hepatic Encephalopathy (MHE)

Patients with liver cirrhosis often exhibit MHE, manifested by mild cognitive impairment, psychomotor slowing, attention deficits, and motor incoordination, which affect their quality of life and life span. MHE also reduces their ability to perform daily tasks such as driving; hinders gainful employment; and increases the risk of home-, work- and traffic-related accidents, resulting in falls, fractures, and hospitalizations. MHE is a serious health, social, and economic problem per se, and it affects several million individuals globally, while also increasing the risk for overt HE [48,49,50,51,52,53,54,55,56,57].

Currently, only two agents have been approved as treatment for HE: lactulose and rifaximin—both acting on the gut microbiome. However, the therapeutic utility of lactulose is limited, and some patients continue to exhibit overt HE while taking rifaximin, which reverses MHE in only 60% of patients [58,59]. This prompted multiple preclinical and early clinical investigations during the last decade to identify the mechanisms responsible for the induction of MHE and to test new drugs with novel mechanisms of action [60,61]. These studies show that the process resulting in MHE involves hyperammonemia and a shift in the immunophenotype and peripheral inflammation with an increase in the activation of some subtypes of CD4 lymphocytes, including Th17, and in the levels of pro-inflammatory cytokines in plasma. Thus, peripheral alterations are transmitted to the brain to induce neuroinflammation, with microglia and astrocyte activation and increased pro-inflammatory factors in the cerebellum and hippocampus. These pro-inflammatory factors activate signal transduction pathways that enhance GABAergic neurotransmission in the cerebellum and hippocampus, resulting in motor incoordination, cognitive impairment, and sedation. Acting on any stage of this process (peripheral inflammation, microglia activation, neuroinflammation, or GABAergic neurotransmission) may reduce sedation and improve cognitive function and motor coordination [61,62,63,64,65,66,67].

Preclinical data from rat models show that a key target that may result in sustained improvements in MHE neurological (cognitive and motor) function is enhanced GABAergic neurotransmission. Hyperammonemia is a main contributor to the enhancement of GABAergic neurotransmission in MHE. The mechanisms involved have been delineated in detail in hyperammonemic rats. Hyperammonemia induces peripheral inflammation and neuroinflammation, with increased activation of microglia and astrocytes in the cerebellum and hippocampus. Activated glial cells produce pro-inflammatory factors, including TNFα, which activates the TNFα-TNFR1-S1PR2-CCL2-BDNF pathway in the cerebellum. This results in the increased activation of the BDNF receptor TrkB in neuronal and glial cells, in turn increasing GABAergic neurotransmission via an increase in GABA synthesis and extracellular GABA levels and the membrane expression of GABA_A_ receptors [66,68,69]. In addition, activated glial cells upregulate the production of allopregnanolone, contributing to the increased GABAergic neurotransmission in a dual autocrine/paracrine manner [70,71]. It has also been reported that increased levels of neurosteroids that enhance GABA_A_ receptor activation, such as THDOC or allopregnanolone, contribute to enhanced GABA neurotransmission in animal models of hyperammonemia and MHE and in cirrhotic patients with HE [34,35,72,73].

Moreover, increased TNFα also enhances GABAergic neurotransmission by increasing the activation of the TNFα-TNFR1-glutaminase-GAT3 pathway. Hyperammonemia increases TNFα and the number of TNFR1 receptors in the membrane, resulting in an increase in the activation of the TNFR1 receptor, which results in increased translocation of the transcription factor NF-κB to the nucleus. This increases the expression of glutaminase, increasing glutamate formation and their extracellular levels. Elevated glutamate increases activation of glutamate transporters and intracellular Na+ content, which reverses the function of the GABA transporter GAT3 in activated astrocytes, resulting in the release and increased extracellular concentrations of GABA [74,75].

GABAergic neurotransmission is enhanced in the cerebellum in vivo, both in animal models [72] and in cirrhotic patients with HE [76]. This increased GABAergic neurotransmission in the cerebellum is responsible for motor incoordination in hyperammonemia and MHE. GABAergic neurotransmission is also increased in the hippocampus, contributing to cognitive impairment [77].

Treatments that reduce GABAergic neurotransmission reverse motor coordination impairment and some aspects of cognitive function in hyperammonemia and MHE. This has been demonstrated in animal models treated with bicuculline, an antagonist of GABA_A_ receptors [72,78,79]; pregnenolone sulphate [80]; or JTE-013, an antagonist of S1PR2, which reduces the enhancement of GABAergic neurotransmission by neuroinflammation [68]. However, these treatments are not useful in clinical practice for different reasons. Bicuculline blocks GABA_A_ receptors and strongly reduces excessive pathological effects, but it may also reduce normal physiological GABAergic neurotransmission, which is necessary for the normal functioning of the brain and can result in anxiety, convulsions, and seizures. Pregnenolone sulphate and JTE-013 were administered intracerebrally, which serves as proof of concept for the mechanism involved but is not applicable in clinical practice.

Flumazenil, a synthetic benzodiazepine antagonist, has been evaluated in several clinical trials aiming to improve HE through an indirect negative allosteric modulatory effect on GABA_A_ receptor function. In a Cochrane meta-analysis, Goh and co-workers [81] found some evidence suggesting a short-term beneficial effect of flumazenil on HE in patients with cirrhosis. Although the interpretation of data is confounded by short-term human exposure to flumazenil, there are potential side effects and modest inverse agonistic effects on GABA_A_ receptors, it may serve to suggest potential benefits of treating cirrhosis and HE individuals with safe and efficacious negative GABA_A_ receptor modulators.

New useful treatments for clinical practice that reduce excessive pathological GABAergic neurotransmission without affecting its normal physiological function are therefore necessary to improve cognitive and motor function in hyperammonemia, MHE, and other neuropsychiatric disorders. This may be achieved using neurosteroids or derivatives [82,83]: for example, the 3β-epimer of allopregnanolone, iso-allopregnanolone, as described above. In a randomized, double-blind study on the efficacy and safety of iso-allopregnanolone (sepranolone) in premenstrual dysphoric disorder (PMDD), sepranolone administered subcutaneously was well tolerated, with preliminary efficacy results indicating an attenuating effect on symptoms, impairment, and distress in women with PMDD [84].

Golexanolone, an experimental drug in clinical development, was further developed to cover GABA_A_-receptor-induced cognitive dysfunction and sedation, and it must be administered orally to comply with chronic administration and achieve a broader range of exposure in humans. As for sepranolone, orally available golexanolone does not act directly on GABA_A_ receptors to reduce their activity as negative allosteric modulators (steroid-NAMs) do, but as discussed previously, it reduces the potentiation of the activation of GABA_A_ receptors via neurosteroid-positive allosteric modulators (steroid-PAM, such as allopregnanolone), including extra-synaptic and synaptic receptor sub-types, such as α5β3γ2L and α1β2γ2L GABAA receptors, respectively. This allows the GABA_A_ receptor’s physiological function to remain intact.

Johansson et al. [47] showed that golexanolone (formerly known as GR3027) reverses motor incoordination assessed in the beam walking test in two models of MHE: rats with chronic hyperammonemia without liver failure and rats with liver failure due to portacaval anastomosis. Golexanolone also improved spatial learning and memory in both models in the Morris water maze and radial maze tests.

In a subsequent study carried out on rats with chronic hyperammonemia and MHE, Mincheva et al. [85] showed that golexanolone also improves short-term memory in the Y maze and object location memory, in addition to motor coordination in the motorater test and some aspects of locomotor gait as assessed in the catwalk test, including stride length, step cycle, and print position.

Moreover, they also analyzed some mechanisms underlying the beneficial effects of golexanolone. Golexanolone reduces neuroinflammation and reverses microglia and astrocyte activation, as assessed morphologically by immunohistochemistry, in the hippocampus and in the molecular layer and white matter of the cerebellum.

The reduction in neuroinflammation is associated with a reversal of the enhanced activation of the TNFα-TNFR1-glutaminase-GAT3 pathway in the cerebellum. Hyperammonemia increases the content of TNFα and the membrane expression of its receptor TNFR1, resulting in increased glutaminase content. This further triggers an increase in the content and membrane expression of the GABA transporter GAT3, for which its function is reversed in hyperammonemia and MHE, increasing extracellular GABA in the cerebellum (see above). Treatment with golexanolone reverses the increases in the content of TNFα, glutaminase, and GAT3 and in the membrane expression of TNFR1 and GAT3 [85].

Moreover, golexanolone also reverses the hyperammonemia-induced activation of the TNFα-TNFR1-S1PR2-CCL2-BDNF pathway in the cerebellum of affected rats, as described previously. Golexanolone reverses the increases in the contents of TNFα, CCL2, TrkB, and GAD67 and the membrane expression of TNFR1, P2X4, and KCC2 [85].

**Figure 2:**
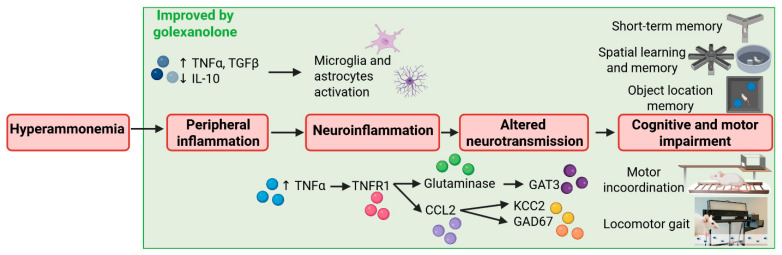
Scheme summarizing the effects of golexanolone on different points of the process resulting in cognitive and motor impairment in rats with hyperammonemia and minimal hepatic encephalopathy. Based on the results of Johansson et al. [47] and Mincheva et al. [85].

It is noteworthy that this study also identified a beneficial effect of golexanolone on peripheral inflammation. Rats with chronic hyperammonemia exhibited increased TNFα and TGFβ plasma levels and reduced anti-inflammatory IL-10 levels. Golexanolone reversed the increase in TNFα and the reduction in IL-10 and, partially, the increase in TGFβ in the plasma of hyperammonemic rats. As described previously, peripheral inflammation plays a key role in triggering cerebral alterations, resulting in cognitive and motor impairment in cirrhotic patients with MHE. This study shows that golexanolone improves cognitive and motor function in hyperammonemia and MHE by acting at multiple points in the pathway linking liver disease to MHE: peripheral inflammation, neuroinflammation, and enhanced GABAergic neurotransmission. These results—summarized in Figure 2 and Table 1—support the hypothesis that golexanolone may have potent beneficial effects in improving cognitive and motor function in cirrhotic patients with MHE via a combination of effects on different stages of the mechanisms resulting in MHE.

The mechanisms mediating the effect of golexanolone on peripheral inflammation should be investigated in future studies. Moreover, additional studies on the effects of golexanolone on other symptoms (sleep alterations, attention deficits, etc.), different animal models of MHE, and liver-disease-associated neurological impairment would also be interesting.

The above studies suggest that since golexanolone improves general pathological mechanisms—such as peripheral inflammation and neuroinflammation—associated with different neurological diseases, it can also be evaluated for the treatment of other pathologies, such as multiple sclerosis or sepsis-associated encephalopathy. In addition, it can be used to understand a possible role of GABAergic neurotransmission in other neurological and inflammatory pathologies. These studies will help our understanding of the interaction mechanisms between GABA neurotransmission and inflammation.

## 3. Golexanolone Clinical Studies in Allopregnanolone Challenge and in Hepatic Encephalopathy

In healthy volunteers, golexanolone exhibited linear, well-tolerated PK in single- and multiple-ascending dose studies (single: up to 200 mg; 100 mg BID ×5 days) [67] (clinical trial registration number: EudraCT 2015-004911-19). In a double-blind, placebo-controlled, three-way crossover allopregnanolone challenge in the same study, oral golexanolone administered 90 min pre-allopregnanolone produced clear CNS target engagement: 30 mg significantly antagonized allopregnanolone-induced reductions in maximal saccadic eye velocity (SEV) in prespecified analyses, and both 3 mg and 30 mg reduced allopregnanolone-induced sedation in responder-focused post hoc analyses. Golexanolone alone exhibited no SEV/sedation effects, consistent with the selective blockade of neurosteroid-mediated GABAA modulation. Although direct CSF measurements were not reported, the pharmacodynamic reversal of allopregnanolone’s CNS effects after oral dosing—together with supportive animal data indicating similar brain and plasma time courses—provides evidence that golexanolone reaches the brain at functionally active levels. Notably, antagonism was observed at allopregnanolone exposures exceeding the concentrations documented in HE brains.

The allopregnanolone challenge study—carried out by Johansson et al.—provided key human proof-of-mechanism data for oral golexanolone, but it was also limited by its use of a small sample of healthy male volunteers, constraining generalizability. Short exposure durations and limited safety data underscore the need for independent, patient-based studies to confirm the clinical relevance and durability of golexanolone’s effects.

Hence, in order to explore the potential benefit of golexanolone in patients, Montagnese et al. [29] performed a pilot phase IIa study (clinical trial registration number: EudraCT 2016-003651-30) in cirrhotic patients with MHE (also named covert hepatic encephalopathy, CHE). In addition to cognitive and motor impairment, these patients also exhibited altered electroencephalogram (EEG) and sleep architecture and quality, sleepiness, and poor attention span, which affect their ability to carry out daily activities [86,87,88]. Montagnese et al. [29] performed a randomized, double-blinded, placebo-controlled study consisting of several stages. First, they studied the safety, tolerability, and pharmacokinetics of golexanolone in cirrhotic patients. Once these parameters were established, they performed an additional study on 45 adult cirrhotic patients with MHE, as determined by abnormal continuous reaction-time (CRT) Index at screening. The patients were treated for 3 weeks with different doses of golexanolone (33 on golexanolone in doses of 10, 40, and 80 mg twice daily; 12 on placebo). In the 3-week efficacy phase, the key endpoints included the following: quantitative EEG; qEEG (mean dominant frequency, MDF; delta + theta/alpha + beta ratio, DT/AB); ESS (Epworth Sleepiness Scale); cognitive tests (CRT stability; animal naming test, ANT); and the psychometric hepatic encephalopathy score (PHES) battery. Via prespecified analyses, golexanolone treatment was associated with directionally favourable changes in EEG—as reflected by MDF (*p* = 0.142), the relative power of theta (*p* = 0.0086), and DT/AB (*p* = 0.021)—and ESS (*p* = 0.047). Cognitively, CRT stability improved, and ANT scores increased in the active arm; the mean PHES improved in the golexanolone group versus minimal changes in the placebo group, although between-group differences did not reach significance (likely due to the short duration and small sample size).

The study by Montagnese et al. provides valuable clinical pilot data, but it is limited by its small sample size, short three-week treatment, and 3:1 randomization ratio, which reduced statistical power and increased site variability.

Thus, although preliminary and subject to certain limitations, early clinical studies indicate that golexanolone demonstrates coherent proof of mechanism and an encouraging preliminary efficacy/safety profile. In healthy volunteers, oral golexanolone exhibited dose-linear, well-tolerated pharmacokinetics and, importantly, reversed allopregnanolone-induced CNS effects (notably saccadic eye velocity reductions and sedation), supporting central target engagement consistent with the negative modulation of neurosteroid-sensitive GABA_A_ receptors. In patients with covert HE, short-term randomized data suggest improvements across select neurocognitive and EEG measures, alongside acceptable safety and PK, providing a clinically relevant extension of human pharmacology. Future studies should address longer, adequately powered, placebo-controlled phase 2b–3 studies with pre-specified primary endpoints for dose–response and exposure–response relationships.

## 4. Golexanolone Reduces Fatigue, Motor Incoordination, and Gait and Improves Cognitive Function in Rats with Bile-Duct Ligation—A Model of Primary Biliary Cholangitis (PBC)

PBC is a chronic autoimmune cholestatic disease with increasing incidence worldwide, and it is characterized by the persistent elevation of cholestatic liver enzymes and circulating bile acids and the presence of antibodies against mitochondria or anti-nuclear antibodies. An intricate interplay involving immune cells, cytokines, and biliary epithelial cells is proposed to underlie the pathogenic mechanisms resulting in the development and progression of PBC [89].

PBC strongly affects health-related quality of life. Many patients with PBC suffer from numerous symptoms, including fatigue and cognitive impairment. Fatigue is the most common and debilitating symptom, affecting more than 50% of the patients, and it strongly reduces PBC patients’ quality of life. In a recent study with a United States cohort of patients with autoimmune liver disorders, PBC most severely impacted health-related quality of life (HRQOL), and this was driven primarily by cognitive symptoms such as tiredness, sleepiness, concentration, and pain [90].

Fatigue is not associated with disease severity, but it is associated with depression, autonomic dysfunction, and sleep disturbances, with no approved or efficacious treatment [89,91,92,93,94]. Fatigue in patients with PBC is associated with an increased risk of death [95,96].

PBC patients may also exhibit cognitive symptoms and problems with respect to concentration and memory (centrally, CNS-mediated fatigue), which seriously impair their quality of life [89,97]. Newton et al. [98] reported that 53% of PBC patients exhibit moderate or severe problems with concentration and/or memory, which were unrelated to liver disease severity. They also found specific cognitive abnormalities with respect to verbal fluency, cognitive processing, attention, visuomotor coordination, and motor and mental speed. Cognitive impairment was associated with autonomic dysfunction and impaired quality of life. Patients with PBC may also exhibit impaired motor function [99].

The only widely approved first-line therapy for PBC is generic ursodeoxycholic acid (UDCA). Second-line Ocaliva^®^ has been withdrawn from the European and US markets. Neither the PPAR agonists Livdelzi^®^ (seladelpar) and Iqirvo^®^ (elafibranor)—which have been conditionally approved for second-line use in PBC in Europe and the USA—nor UDCA or other current therapies for PBC have a meaningfully beneficial effect on behavioural symptoms [100]. Since no approved treatments exist for these symptoms, new treatments are, therefore, necessary and part of the development strategies for improving symptoms such as fatigue or cognitive impairment.

Fatigue in PBC seems to have both cerebral and peripheral components. Peripheral inflammation contributes to fatigue in patients with cancer, multiple sclerosis, Parkinson’s disease, or rheumatoid arthritis, and likely in PBC [101,102]. As described above for hyperammonemia and MHE, peripheral inflammation could also contribute to central fatigue by inducing neuroinflammation [103,104,105], which alters neurotransmission, resulting in altered brain function (the liver–brain axis). Morris et al. [105] proposed that enhanced pro-inflammatory factors alter neurotransmission, resulting in the induction of fatigue. The alterations in neurotransmission are reflected in brain function alterations that can be observed by magnetic resonance imaging or PET scan studies [105,106]. McDonald et al. [99] showed that PBC patients indeed exhibit brain dysfunction. They used transcranial magnetic stimulation to analyze cortical inhibitory and excitatory neurotransmission. They found that both intra-cortical inhibition (ICI) and facilitation (ICF), as well as their balance, are altered in PBC patients, resulting in decreased central activation, which may be wholly or partially responsible for sleep disturbances, fatigue, and motor and cognitive symptoms. This study suggests that the impaired balance between inhibitory (GABAergic) and excitatory (glutamatergic) neurotransmission may contribute to fatigue, cognitive deficits, and other symptoms in PBC patients. A role for altered GABA neurotransmission in fatigue induction has already been proposed. Patients with chronic fatigue symptoms and PBC patients with fatigue (but not those without fatigue) exhibit increased plasma levels of allopregnanolone, an endogenous neurosteroid-PAM (discussed above) that potentiates GABA_A_ receptor activation [35,107]. Wetten et al. [108] showed that increased levels of serum allopregnanolone are associated with cognitive and emotional symptoms in pre-cirrhotic PBC patients and proposed that reducing the activation of GABA_A_ receptors by neurosteroids may be a target for improving emotional and cognitive symptoms in PBC.

Arenas et al. [31] tested this possibility in a rat model of PBC, performing a preclinical modelling study to inform potential intervention studies in PBC patients. Rodents with bile-duct ligation (BDL) are the most extensively used model of cholestasis. Rats with bile-duct ligation exhibit cholestasis and fatigue, in addition to cognitive and motor impairment [43,64,91,109,110,111].

Arenas et al. [31] treated BDL rats with golexanolone, a GABA_A_-receptor-modulating steroid antagonist that reduces the potentiation of GABA_A_ receptors by allopregnanolone in animal models and humans (see above). Moreover, they assessed whether golexanolone reverses the key symptoms affecting PBC patients: fatigue and cognitive and motor impairment. Rats with BDL exhibited increased fatigue, as analyzed via a treadmill test, both at 2 and 5 weeks after BDL surgery. Treatment with golexanolone completely reversed this effect. BDL rats treated with golexanolone did not show increased fatigue compared to control rats 2 or 5 weeks after surgery.

BDL rats also exhibited impaired short-term memory in the Y maze test. Golexanolone completely reversed this cognitive impairment [31].

BDL rats also exhibited motor incoordination in the motorater test, with increased wrong foot placement and run errors and altered locomotor gait, as analyzed in the catwalk test. Golexanolone normalized motor coordination and locomotor gait, reversing these changes.

Golexanolone, therefore, completely reversed the main symptoms affecting PBC patients: fatigue and cognitive and motor impairment.

Arenas et al. [31] also assessed whether improvements in the behavioural symptoms introduced by golexanolone are associated with peripheral inflammation and neuroinflammation improvements, which are key factors in the development and maintenance of behavioural symptoms, as summarized above.

BDL rats exhibited peripheral inflammation, with increased pro-inflammatory cytokine plasma levels, including TNFα, IL-18, IL-17, IL-6, HMGB1, IFNƴ, and CCL2, and reduced anti-inflammatory IL-10 levels. Treatment with golexanolone improves several key pro-inflammatory cytokines, such as TNFα, IL-18, IL-17, and IL-6, but it did not affect other aspects of peripheral inflammation, such as IFNƴ, CCL2, or IL-10. These data clearly show that golexanolone has an important peripheral anti-inflammatory effect in BDL rats. This reinforces the idea already proposed for rats with hyperammonemia and MHE (see above) that, in addition to the direct effects on GABAergic neurotransmission, golexanolone also affords beneficial effects in hyperammonemia, MHE, PBC and likely other pathologies by reducing peripheral inflammation. It has been shown that peripheral inflammation is a key trigger of neuroinflammation and behavioural symptoms in these and other pathologies. The dual effect on peripheral inflammation and GABAergic neurotransmission endows golexanolone with a strong therapeutic potential in a wide variety of pathologies.

A key mediator between peripheral inflammation and the alterations in neurotransmission, fatigue, and cognitive and motor function is glial activation and neuroinflammation [36,64,65,112,113,114,115,116].

BDL rats sacrificed at 6 weeks after surgery exhibited microglia activation in the molecular layer of the cerebellum, as assessed via the morphological analysis of Iba1-stained cells, and a loss of astrocytes stained with GFAP, indicating late-stage astrocyte damage. Golexanolone treatment reversed microglia activation and the loss of GFAP staining, indicating improvements with respect to glial activation and damage [31].

Glial activation contributes to neuroinflammation, altered neurotransmission, and fatigue and cognitive and motor impairment, by increasing pro-inflammatory factor levels that trigger the activation of pathological transduction pathways, as discussed in the previous section on hyperammonemia and MHE.

BDL rats show increased levels of pro-inflammatory TNFα, IL-1β, IL-6, and glutaminase in the cerebellum, and treatment with golexanolone completely reversed the increase in these proteins [31]. The beneficial effects of golexanolone on glial activation, neuroinflammation, and the content of these proteins likely play a key role in the mechanisms by which it improves fatigue and cognitive and motor function.

Golexanolone also improved some alterations in GABAergic system in the cerebellum of BDL rats, reversing the increase in the content of the GABA-synthesizing enzyme GAD67 and the β3 subunit of GABA_A_ receptors and reversing the reduction in GABA transporter GAT1 contents [31].

**Figure 3:**
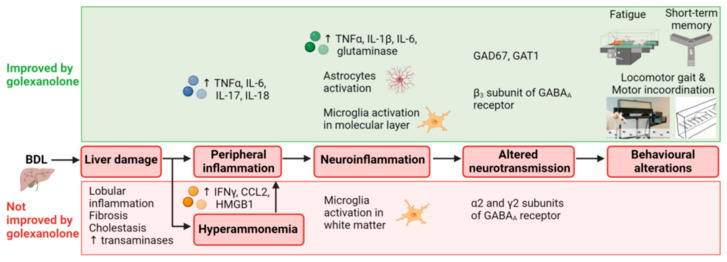
Scheme summarizing the effects of golexanolone on different stages of the process resulting in fatigue and cognitive and motor impairment in BDL rats. From Arenas et al. [31].

The effects of golexanolone on the different stages of the process by which liver damage in BDL rats results in fatigue and cognitive and motor impairment are summarized in Figure 3 and Table 2.

The effects of golexanolone on neuroinflammation and neurotransmission in other brain areas would be interesting for future studies. Further research is needed to understand the time course and the effects of golexanolone on the onset of cholestasis and bile acid regulation. It is also necessary to investigate how the modulation of GABA_A_ receptors via allopregnanolone, which is blocked by golexanolone, modulates peripheral cytokine levels and induces neuroinflammation.

The preclinical modelling study of Arenas et al. [31] reported that golexanolone is a promising therapeutic tool for improving the debilitating symptoms that affect the quality of life of patients with PBC: fatigue and cognitive impairment (central fatigue) and also motor impairment. In 2023, the U.S. Food and Drug Administration (FDA) granted the Orphan Drug Designation to golexanolone for the treatment of PBC.

As central fatigue is a main problem in other diseases and pathological conditions associated with inflammation and autoimmune diseases—such as multiple sclerosis, rheumatoid arthritis, or fibromyalgia [117]—the benefit of golexanolone treatment could be tested in these contexts, and the observations could be used to use golexanolone as a more general treatment. However, further studies are needed to thoroughly analyze potential side effects in different situations.

## 5. Golexanolone Reduces Glial Activation and Improves Motor Incoordination, Some Gait Alterations, Fatigue, Anxiety, Depression, and Short-Term Memory in a Rat Model of Parkinson’s Disease (PD)

PD is the second most common neurodegenerative disorder after Alzheimer’s disease. PD typically develops between 55 and 65 years, with a prevalence of 1–2% in people over 60 years, increasing to 3.5% at 85–89 years. PD affects over 6 million people worldwide and is expected to affect more in the upcoming decades [118,119,120]. PD imposes an important economic burden on health systems. Yang et al. [121] report at least one million affected individuals in the United States, with annual costs of USD 52 billion.

PD is a progressive disorder characterized by motor symptoms, including impaired motor coordination and locomotor gait, bradykinesia, freezing of gait, rigidity, resting tremor, and impaired postural reflexes. PD patients also exhibit a variety of non-motor symptoms, including neuropsychiatric symptoms, autonomic and enteric dysfunction, and sleep disorders. For many patients, non-motor symptoms contribute immensely to disability and often impact daily activities more than motor symptoms do. The burden of non-motor symptoms can define a patient’s health-related quality of life. Non-motor symptoms in PD patients include mild cognitive impairment and executive dysfunction, fatigue, anxiety, and depression, all of which strongly impair their quality of life [122,123,124,125,126,127,128]. There are currently no treatments approved for sleep comorbidities or cognitive impairment in Parkinson’s disease.

Similarly, there are currently no drugs available for slowing the progression of PD, representing a major unmet need. L-DOPA (levodopa) is considered the most effective treatment for PD patients [129]. L-DOPA alleviates the main motor symptoms of PD, but it provides limited relief and has side effects. Most patients develop uncontrollable abnormal involuntary movements, which are known as L-DOPA-induced dyskinesias. Patients often exhibit L-DOPA-refractory symptoms, such as postural instability and gait difficulties, including falls and freezing of gait. Moreover, L-DOPA does not improve non-motor symptoms. This indicates that novel treatments to improve these L-DOPA-refractory motor and non-motor symptoms—in addition to slowing the progression of PD—are needed [124,130,131].

A promising new therapeutic drug for improving both motor and non-motor symptoms without inducing dyskinesia is golexanolone [30,132,133]. As previously described, golexanolone reduces GABAergic neurotransmission and improves glial activation, neuroinflammation, and cognitive and motor impairment in rats with hyperammonemia, minimal hepatic encephalopathy, and bile-duct ligation [31,47,85]. Enhanced GABAergic neurotransmission, glial activation, and neuroinflammation also play key roles in the pathogenesis of PD (see below). This prompted Izquierdo-Altarejos et al. [30] to assess the beneficial effects of golexanolone in a rat model of PD.

The activation of microglia and astrocytes, and subsequent neuroinflammation, plays a key role in PD [134,135,136]. Kam et al. [134] propose that microglia and astrocyte dysfunction are involved in PD pathogenesis and that a better understanding of microglia and astrocyte roles in PD may provide insights into neurodegeneration and novel therapeutic approaches for PD. Furthermore, neuroinflammation and α-synuclein pathologies are interconnected in a vicious cycle, each exacerbating the other [137,138]. Chen et al. [136] propose that this knowledge could also provide a more reliable diagnosis of PD.

The motor symptoms of PD are the result of depletion of dopamine in the substantia nigra and striatum due to the loss of dopaminergic neurons in the SN, that results in a strong reduction in tyrosine hydroxylase (TH), the enzyme that synthesizes dopamine [139,140,141]. However, Heo et al. [142] reported that a relevant part of TH loss is not due to the loss of dopaminergic neurons but a result of the repression of neuronal TH expression mediated by excessive GABA_A_ receptor activation. A contribution of enhanced GABAergic neurotransmission to the pathogenesis of PD has been proposed by Lemos et al. [143], Muñoz et al. [144], and Heo et al. [142].

In animal models of PD, activated astrocytes in SN exhibit increased levels of GABA, which is released into extracellular fluid, resulting in an aberrant tonic inhibition of dopaminergic neurons; this is mediated via the enhanced activation of GABA_A_ receptors. This over-activation of GABA_A_ receptors inhibits TH expression, resulting in the appearance of “dormant neurons” that express dopamine decarboxylase (DDC) but not TH [142]. This reduction in TH results in a subsequent decrease in dopamine, and motor symptoms consequently follow. Heo et al. [142] also showed that the expression of TH in “dormant neurons” may be restored by blocking GABA_A_ receptors, and this was associated with improvements in motor symptoms. Based on these data, Izquierdo-Altarejos et al. [30] hypothesized that golexanolone could also induce beneficial effects in rats with PD by reducing GABA_A_ receptor activation and reducing glial activation and neuroinflammation. They used the unilateral 6-OHDA rat model of PD and administered golexanolone starting 4 weeks after surgery to inject 6-OHDA, when brain damage is already quite advanced.

The 6-OHDA rats exhibited fatigue in the treadmill test; anxiety in the open field test; anhedonia, a symptom of depression, in the sucrose preference test; and impaired short-term memory in the Y-maze test. The golexanolone treatment improved all these non-motor symptoms, completely reversing anxiety, anhedonia, and short-term memory impairment. Moreover, fatigue was almost completely reversed [30].

The 6-OHDA rats also exhibited motor symptoms, with motor incoordination assessed in the motorater and in the catwalk test, in which a decreased regularity index was observed in these rats. Golexanolone completely reversed motor incoordination.

The 6-OHDA rats also exhibited impaired locomotor gait, as analyzed in the catwalk test. In the 6-OHDA rats, some of the affected parameters include an increase in the initial dual stance and duty cycle and a reduction in swing percentage. These parameters reflect alterations in freezing of gait, postural instability, and bradykinesia, and they were completely improved by the golexanolone treatment. Other parameters of locomotor gait, such as stand, also reflected that bradykinesia was significantly but not completely improved by the golexanolone treatment starting 4 weeks after surgery [30].

One main problem with the current L-DOPA treatment is the induction of dyskinesia, which strongly impairs the quality of life of the patients (see above). Izquierdo-Altarejos et al. [30] reported that golexanolone treatment does not induce dyskinesia. Golexanolone may, therefore, be a very beneficial treatment that improves many motor (incoordination, bradykinesia, freezing of gait, and postural instability) and non-motor (fatigue, depression, anxiety, and memory) symptoms that strongly impair the quality of life and daily functioning of PD patients without inducing dyskinesia side effects.

Izquierdo-Altarejos et al. [30] also analyzed the effects of golexanolone on key aspects of the pathological mechanisms involved in PD, 5 and 10 weeks after 6-OHDA injection surgery.

As described above, microglia and astrocyte activation drive neuroinflammation and play a key role in the pathogenesis of PD. The activation of microglia is usually reflected in a change in morphology, with a reduction in the area and perimeter of microglia cells as analyzed by immunohistochemistry using anti-Iba1. Rats treated with 6-OHDA show the activation of microglia in the striatum, as microglial cells exhibited a reduced perimeter, as analyzed by immunohistochemistry. Golexanolone treatment starting 4 weeks after surgery reverses these morphological changes at 5 weeks but not at 10 weeks post-surgery.

The 6-OHDA rats also exhibited the activation of astrocytes, as reflected by an increase in GFAP stained area in the stratum 5 weeks after surgery, and this is reversed by golexanolone.

As described above, a key contributor to the pathogenesis of PD is the loss of TH. The rats treated with 6-OHDA exhibit a reduction in TH content in the striatum 5 and 10 weeks post-surgery. Treatment with golexanolone from week 4 after surgery reduced TH loss at 5 but not at 10 weeks post-surgery.

A noteworthy finding reported by Izquierdo-Altarejos et al. [30] is that α-synuclein contents are increased in the striatum of 6-OHDA rats in the long term (10 weeks) but not short term (5 weeks) post-surgery, as assessed via both immunohistochemistry and Western blotting. This increase in α-synuclein at 10 weeks most probably follows the increase in neuroinflammation and is completely prevented by golexanolone.

In a classical 6-OHDA rat model, α-synuclein expression is not intrinsically induced. The model’s hallmark is dopaminergic neuron loss and motor asymmetry, not synucleinopathy. However, although the 6-OHDA model does not intrinsically produce Lewy-type α-synuclein pathology, measurable increases in α-synuclein (mRNA/protein, sometimes pSer129) are observed, especially where glial activation and stress pathways are prominent [145,146,147,148]. Mechanistically, pro-inflammatory cytokines (e.g., TNF-α) promote α-synuclein propagation/accumulation, supporting a feedback loop where lesion-triggered inflammation can secondarily elevate α-synuclein [149].

The data reported by Izquierdo-Altarejos et al. [30] demonstrate that golexanolone treatment improves motor and non-motor symptoms in the 6-OHDA rat model of PD via the reduction in microglia and astrocyte activation, in the loss of TH, and the decrease in α-synuclein.

The same group later studied—in more detail—the effects of golexanolone on microglia and astrocyte activation in the substantia nigra and striatum. In many pathological contexts, astrocyte activation is a consequence of previous pro-inflammatory microglia activation, which releases pro-inflammatory factors such as TNFα, IL-1α, and HMGB1 that induce the activation of astrocytes, increasing their vimentin and S100B contents [150,151,152,153].

Mincheva et al. [133] used the same 6-OHDA PD rat model but started treatment with golexanolone at 1 week instead of 4 weeks post-surgery; they analyzed the effects of 6-OHDA injections and golexanolone treatment on microglia and astrocyte activation and their possible interplay 3 and 9 weeks post-surgery. At 3 weeks after surgery, the 6-OHDA rats already exhibited microglia activation both in the SN and striatum, as reflected by the decrease in area and perimeter of Iba1-stained cells. The golexanolone treatment reversed this microglia activation.

As an additional parameter reflecting pro-inflammatory microglia activation, Mincheva et al. [133] also analyzed the content of TNFα in Iba1-labelled microglia cells by double immunofluorescence. Rats treated with 6-OHDA exhibited an increased number of TNFα-positive microglia in both the SN and the striatum. Golexanolone treatment reversed this increase, indicating a reduction in pro-inflammatory microglia activation.

At 3 weeks post-surgery, 6-OHDA rats showed increased levels of TNFα and IL-1α both in the SN and the striatum, and HMGB1 levels were increased in the striatum. This provides evidence that the production and release of these pro-inflammatory factors are increased in activated microglia. Golexanolone treatment reversed the increase in TNFα and IL-1α levels in the SN, and HMGB1 levels were reversed in the striatum.

The increase in these pro-inflammatory factors is associated with astrocytes activation in both the SN and the striatum, as reflected in the increased area stained with anti-GFAP and the increased content of vimentin and S100B. The golexanolone treatment reversed the astrocytes’ activation, likely due to the reduction in TNFα, IL-1α, and HMGB1 levels [133].

**Figure 4:**
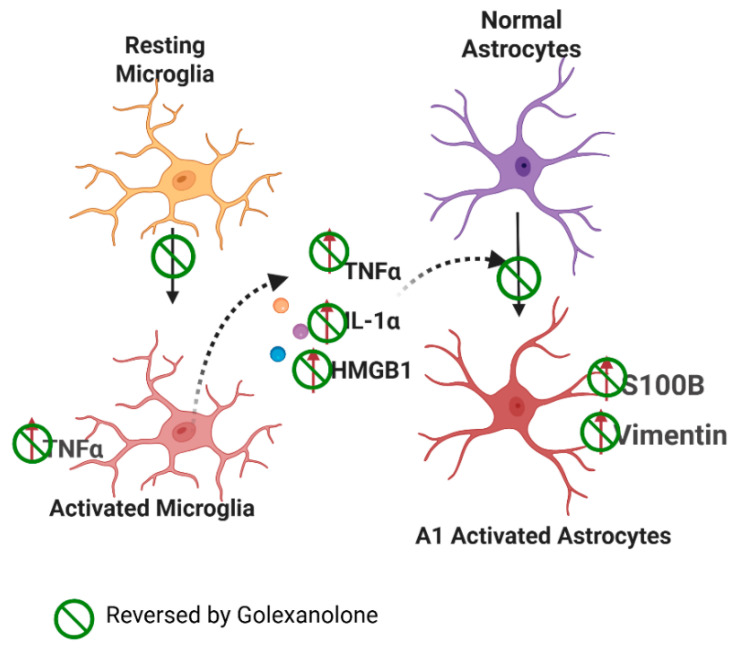
Proposed mechanism of glial activation in the PD model. Effects of Golexanolone. In rats with unilateral injection of 6-OHDA, microglia exhibit a pro-inflammatory activation state in the SN and striatum, characterized by a more amoeboid morphology and increased TNFα contents. Pro-inflammatory microglia releases TNFα, IL-1α, and HMGB1, which induce astrocyte activation, transitioning to a pro-inflammatory A1 form, that exhibits increased vimentin and S100β, in addition to increased GFAP levels. Golexanolone treatment produces a sustained reduction in all these pro-inflammatory factors in SN, reversing microglia and astrocyte activation both at 3 and 9 weeks post-induction of the model, thus reducing neuroinflammation in the 6-OHDA-injected rats. The effects of golexanolone are indicated by 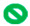. From Mincheva et al. [133].

Similar results were obtained at 9 weeks post-surgery. Rats treated with 6-OHDA exhibited increased microglia activation, as reflected in the reduced area and perimeter and increased TNFα content. This was associated with increased TNFα, IL-1α, and HMGB1 levels and astrocyte activation in SN but not in the striatum. Golexanolone reversed these pathological effects, and the entire process is summarized in Figure 4 and Table 3.

These data suggest that golexanolone reduces microglia activation and astrocyte activation mediated by factors released from pro-inflammatory microglia. The data also show that earlier intervention supports sustained neuroinflammation improvements. New therapeutic options for PD have been proposed, based on mitigating neuroinflammation and specifically aimed to reduce glial activation [154,155,156,157,158]. The results reported by Izquierdo-Altarejos et al. [30] and Mincheva et al. [133] indicate that golexanolone is a highly promising treatment for PD by reducing glial activation and associated neuroinflammation.

Notably, golexanolone reverses microglia activation and completely abolishes the upregulation of α-synuclein protein expression in the 6-OHDA rat model, suggesting that it may disrupt the vicious cycle linking inflammation to α-synuclein [30,133].

These preclinical studies with golexanolone also support the relationship between neuroinflammation, GABAergic neurotransmission mediated by GABA_A_ receptors, and dopamine loss. Further mechanistic research on this relationship is needed to identify—in detail—the mechanisms involved in the pathogenesis of Parkinson’s disease in order to improve diagnosis and therapeutic strategies. The mechanisms underlying golexanolone’s effect on α-synuclein, its potential impact on dopamine D1 and D2 receptors, and the alternative pathways they activate—implicated in Parkinson’s disease pathophysiology—represent important areas for future research.

Studies in other animal models of PD will be useful for supporting these findings and validating the therapeutic effect of golexanolone treatment.

## 6. Conclusions and Future Perspectives

GABA_A_-receptor-modulating steroid antagonists (GAMSAs), such as golexanolone (previously referred to as GR3027), are a unique pharmacological class of compounds that block the effect of steroid-PAMs without disturbing normal GABA receptor functions. This feature renders GAMSAs important tools for both research and therapy, as they can separate steroid modulation from basic GABAergic transmission. The data summarized in this study support the hypothesis that golexanolone is a promising therapeutic tool for treating diverse disorders associated with peripheral inflammation, neuroinflammation, and GABAergic neurotransmission. Studies to date demonstrate beneficial effects on neurological and functional impairments in preclinical models of HE, PBC, and Parkinson’s disease, including the reversal of fatigue, anxiety, and depression, as well as improvements in cognitive function, motor coordination, and locomotor gait. Golexanolone improves these abnormalities by reversing the mechanisms underlying both peripheral inflammation and neuroinflammation, which drive these behavioural abnormalities via altered GABA_A_ receptor neurotransmission.

The data show that golexanolone reverses microglia and astrocyte activation, key triggers of neuroinflammation and its subsequent detrimental effects on neurotransmission and neurological and behavioural functions. Glial activation is involved in many neurological and neurodegenerative diseases (e.g., multiple sclerosis, Parkinson’s disease, Alzheimer’s disease, etc.) and in the cognitive and motor impairments associated with chronic inflammatory diseases, such as SLE, rheumatoid arthritis, or type 1 diabetes. Therefore, it is likely that golexanolone can introduce beneficial effects in many of these disorders by inducing a general anti-inflammatory effect—both in the brain and in peripheral blood. Further studies that confirm this possibility could provide the basis for extending the therapeutic use of golexanolone to a wide range of pathologies involving sustained peripheral inflammation and neuroinflammation.

The key areas for future research include the mechanisms by which golexanolone reverses microglia activation—as this may inform its use in other disorders—and, of course, clinical studies that establish its benefit in humans. In addition, research on the mechanisms mediating the beneficial effects of golexanolone has, to date, focused on neuroinflammation and related pathways, but other possible mechanisms and affected pathways—such as neurotransmitter receptors and associated pathways or neurodegenerative or neurotrophic pathways—can be studied in future research.

The key unknowns include a demonstration of the effects on central fatigue (using objective vigilance measures); identification of translational biomarkers linking neurosteroid modulation to clinical improvement; and assessment of the long-term impact on daily functioning and quality of life. Establishing minimal clinically important differences for fatigue and cognition endpoints will be critical for regulatory validation. For PD, the main unknowns include the absence of human efficacy data for EDS and vigilance, potential interaction effects with dopaminergic therapies, and identification of translational biomarkers that connect neurosteroid activity to wakefulness regulation. Human studies are needed to establish dose–response relationships, tolerability, and sustained efficacy.

## Figures and Tables

**Table 1:** Effects of Golexanolone on several sequential pathological mechanisms and neurological outcomes in rats with hyperammonemia and minimal hepatic encephalopathy.

Pathological Mechanism or Neurological Outcome	Effects of Golexanolone		
**Peripheral Inflammation**	Reduces TNFα and TGFβ plasmatic levels	Increases IL-10 levels in plasma	
**Neuroinflammation**	Reduces microglia and astrocyte activation in cerebellum and hippocampus	Reduces TNFα and CCL2 levels in cerebellum	Reduces TNFR1 and P2X4 membrane expression in cerebellum
**Neurotransmission**	Normalizes GAT3 and KCC2 membrane expression in cerebellum	Normalizes GAD67 content in cerebellum	Normalize GABA_A_-β3 content in cerebellum
**Motor Function**	Improves locomotor gait	Improves motor coordination	
**Cognitive Function**	Improves short-term spatial memory	Improves spatial learning and memory	

Effects of golexanolone on different stages of the process resulting in cognitive and motor impairment in rats with hyperammonemia and minimal hepatic encephalopathy. Based on the results of Johansson et al. [47] and Mincheva et al. [85].

**Table 2:** Effects of golexanolone on several sequential pathological mechanisms and neurological outcomes in bile-duct-ligated rats.

Pathological Mechanism or Neurological Outcome	Effects of Golexanolone		
**Peripheral Inflammation**	Reduces TNFα, IL-6, IL-17, and IL-18 plasmatic levels		
**Neuroinflammation**	Reduces microglia and astrocyte activation in cerebellum	Reduces TNFα, IL-1β, IL-6, and glutaminase contents in cerebellum	Increases IL-10 content in cerebellum
**Neurotransmission**	Reduces GAD67 content in cerebellum	Increases GAT-1 content in cerebellum	Decreases GABA_A_-β3 content in cerebellum
**Motor Function**	Improves locomotor gait	Improves motor coordination	Reduces fatigue
**Cognitive Function**	Improves short-term spatial memory		

Effects of golexanolone on different stages of the process resulting in fatigue and cognitive and motor impairment in BDL rats, a model of PBC. From Arenas et al. [31].

**Table 3:** Effects of Golexanolone on neuroinflammation, neurotransmission, and neurological impairment in a 6-OHDA rat model of Parkinson’s disease.

Pathological Mechanism or Neurological Outcome	Effects of Golexanolone		
**Protein Aggregation**	Reduces α-synuclein content		
**Microglia Activation**	Reduces ameboid morphology of activated microglia in substantia nigra and striatum	Reduces content of TNFα in microglia of substantia nigra and striatum	Reduces TNFα, IL-1α, and HMGB1 levels in substantia nigra and striatum
**Astrocyte Activation**	Decreases GFAP content in substantia Nigra and striatum	Reduces vimentin and S100B levels, markers of pro-inflammatory astrocytes A1	
**Neurotransmission**	Increases TH content in striatum		
**Motor Function**	Improves locomotor gait	Improves motor coordination	Reduces fatigue
**Cognitive Function**	Improves short-term spatial memory	Reduces anxiety and anhedonia (depression symptoms)	

Based on the results of Izquierdo-Altarejos et al. [30] and Mincheva et al. [133].

## Data Availability

No new data were created or analyzed in this study. Data sharing is not applicable to this article.

## Abbreviations

The following abbreviations are used in this manuscript:

CNS	Central nervous system;
NAMs	Negative allosteric modulators;
PAMs	Positive allosteric modulators;
GAMSAs	GABAA-Receptor-Modulating Steroid Antagonists;
THDOC	Tetrahydrodeoxicorticosterone;
DHEAS	Dehydroepiandrosterone sulphate;
PD	Parkinson’s disease;
HE	Hepatic encephalopathy;
MHE	Minimal hepatic encephalopathy;
PBC	Primary biliary cholangitis;
MoCA	Montreal Cognitive Assessment;
PHES	Psychometric Hepatic Encephalopathy Score;
EEG	Electroencephalogram;
UDCA	Ursodeoxycholic acid;
BDL	Bile duct ligation;
FDA	Food and Drug Administration;
ODD	Orphan drug designation;
TH	Tyrosine hydroxylase;
6-OHDA	6-Hydroxydopamine.
